# Evaluation of Antibacterial Activity of Pine Tar on Periodontal Pathogenic Bacteria: An In Vitro Study

**DOI:** 10.4314/ejhs.v30i6.17

**Published:** 2020-11

**Authors:** Eman Ali Alqahtani, Mohamed Fadul A Elagib, Rawan Hamad Al-Yami, Alanoud Saeed Abu Hatlah, Amel I Faragalla, Raghvendra Reddy

**Affiliations:** 1 Dentist, Abha, Kingdom of Saudi Arabia; 2 Assistant Professor, College of Dentistry, King Khalid University, Abha, Kingdom of Saudi Arabia

**Keywords:** anti-bacterial, anti-microbial, periodontitis, pine tar

## Abstract

**Background:**

Periodontal pathogens play an important role in etiology and pathogenesis of periodontitis. Microbiological examination of sub gingival plaque is used at the present time in etiological research as well as in clinical treatment of periodontitis to select the appropriate antibiotic agent if indicated. Pine tar has been used for the treatment of various skin diseases. So the study was done to evaluate the effect of Pine Tar oil on bacteria isolated from periodontitis patients.

**Methods:**

Plaque samples from volunteer patients were collected using sterile paper points. Robertson's Cooked Meat (RCM) medium was used for the transportation and cultivation of aerobic, microaerophilic, and anaerobic microorganisms.

**Results:**

The result suggests the use of Pine tar oil for topical application in periodontal diseases. Disc diffusion analysis was sufficient enough to illustrate that 75 µl tar oil solution produced growth inhibition of microbial strains.

**Conclusion:**

Pine tar oil has become one of the important areas of research both in pharmaceutical and periodontal research, hence in vivo studies has to be carried out with various form of pine tar.

## Introduction

The advent of bacterial resistance in every new generation of its reproduction cycle compels scientific community to develop new subjects of antimicrobials agents in order to mitigate the rapid spread of serious infection. A steady flow of antibiotics provide a viable option to treat bacterial infection (1). The increase of bacterial resistance, have pecked the scientific world to invest in alternative sources of anti-microbial agents with minimal or zero side effects. Pine tar is a potent anti-septic agent and also reported to have anti-microbial properties. It is extracted from pine wood carbonization after distillation by extreme heat. Also, pine tar has been in traditional medicine practice for more than 2000 years in the treatment of skin ailment. In addition, Pin tar has been reported for its anti-pyretic, anti-inflammatory, anti-fungal, antipruritic, astringent, keratoplastic, cytostatic, antibacterial, and anti-microbial effect. Its chemical composition is extremely complex, constituting of more than thousand single molecules, primarily aromatic hydrocarbons, tar acids and tar bases (2,3). The principal constituents of pine tar include turpentine, resin, guaiacol, creosol, methylcreosol, phenol, phlorol, toluene, xylene and other hydrocarbons. And because of these properties pine tar has been used to treat eczema and psoriasis (4).

In many cases, the pine tar is used along with coal tar leading to poor results and misjudged for its mechanism of action. Pine tar is not pharmacologically standardized because of its inherent chemical complexity. Besides, the specific therapeutic activity of its components is also not known (4). Further, studies based on extraction, isolation and characterization of bioactive molecules present in pine tar, following a series of *in-vitro* and *in-vivo* studies is an essential probe to know its mechanism of action. Against this background, the present study was aimed to evaluate the effect of Pine Tar oil on bacteria isolated from subgingivial plaque samples of patients with periodontitis.

Periodontal pathogens play an important role in etiopathogenesis of periodontitis; although they are not the only deciding factor, their absence in the periodontal pocket indicates more stability and better prognosis. Microbiological examination of sub gingival plaque is used at the present time in etiological research as well as in clinical treatment of periodontitis to select the appropriate antibiotic agent if indicated. Examples of such conditions are aggressive periodontitis, advanced chronic periodontitis, refractory periodontitis, moderate and advanced chronic periodontitis in combination with systemic diseases or conditions that affect the immune system (5).

Pine tar was also known in ancient Greece (6). In fact, the use of pine tar in medicine was first described by Hippocrates more than 2000 years ago, and has probably been produced in Scandinavia since the Iron Age (7,8). Pine tar is a dark brown or nearly black viscous semi-liquid, which is denser than water and has a characteristic empyreumatic odour and a peculiar sharp taste. Many modifications are done to tar preparations, to increase their acceptability like handling the messy application, masking its odour and consideration of cloth staining (7). Today, it is available in various formulations including a gel, lotion, oil, soap-free bar and solution containing up to 2.3% w/w pine tar (8). Four sources of tar have been used for therapeutic treatments; wood (wood tar), bitumen (shale tar), petroleum (petroleum tar) and coal (coal tar). There are two kinds of wood tars; made either from trees with a high content of resin (pine and juniper), or from hardwood trees (birch and beech). Pine tar also known as tar, alquitran vegetal, pix liquida, stockholm tar in commerce (9).

The use of medicinal tar for dermatologic disorders dates back to the ancient times. Although coal tar is utilized more frequently in contemporary dermatology and medicine, wood tars have also been widely employed (7). Pine tar has been used in topical preparations to relieve itching and inflammation associated with a range of skin conditions such as eczema, psoriasis, chronic lichen simplex, seborrhoeic dermatitis and scalp psoriasis, sunburn, nappy rash, prickly heat, hives, chicken pox, insect bites, anal and genital itching including jock itch, and other dry, itchy, flaky or inflamed skin conditions (10). Pine tar has either been used alone or in combination with other medications, phototherapy, or both. Occupational studies have demonstrated the carcinogenicity of tar; however, epidemiologic studies do not confirm similar outcomes when used topically (7). Therefore it is widely use in the dermatological sector and now its application is being considered in other fields of medicine owing to its great biological benefits.

## Methods

Experimental in vitro study was done in College of Dentistry, King Khalid University Dental Clinics, male and female campuses. The institutional review board of the university approved the study, and gave the registration number IRB/KKUCOD/ETH/2019-20/063. The study was according to the ethical principles of the World Medical Association Declaration of Helsinki (2013). Volunteers were informed about the study protocol, and written consent was obtained before they participated in the study. Plaque samples were collected using sterile paper points. Robertson's Cooked Meat (RCM) medium was used for the transportation and cultivation of aerobic, microaerophilic, and anaerobic microorganisms. It is also known as “cooked meat broth” as it contains pieces of fat free minced cooked meat of ox heart and nutrient broth. Collected samples were labeled and transported to the laboratory for processing.

Samples were processed for culture by standard conventional methods using Mueller Hinton agar medium (Oxoid Ltd., Basingstoke, and Hampshire, United Kingdom) as the primary isolation medium while Blood agar plates were used for other analysis and examination. Preliminary bench identification was done by standard phenotypic tests. Following preliminary identification, confirmation of isolates was done at the microbiology laboratory using the VITEK - 2 identification system (BioMerieux, France) according to manufacture criteria.

*Porphyromonasgingivalis, P.intermedia, Aggregatibacteractinomycetemcomitans* were cultured as per the standard protocol. The organisms were incubated with pine tar gel and incubated aerobically for 24 hours. Afterward, results were read as inhibition of bacterial growth by the pine tar. An inhibition zone of about 12–13 mm around the 75 µl well was observed in all the four species in the agar plates.

Process included bacterial isolation, enumeration of microorganisms, initial identification of isolated microorganisms, and identification of isolated microorganisms using VITEK. The determination of the MIC of Tar and assays for antimicrobial activity of Tar oil has been done. Antimicrobial activity was assessed by measuring the observed inhibition zone diameter.

## Results

The result of disc diffusion analysis was sufficient enough to illustrate that 75 µl tar oil solution produced growth inhibition of microbial strains. Serial dilution of 1/10, 1 /100, and 1/100 of tar oil were conducted against *P. gingivalis* and *P. intermedia, A. actinomycetemcomitans* species. The inhibitory results were similar to the original tar oil for 1.10 and 1.100 dilutions. However, no inhibition was noticed with the 1/1000 dilution

**Figure 1:**
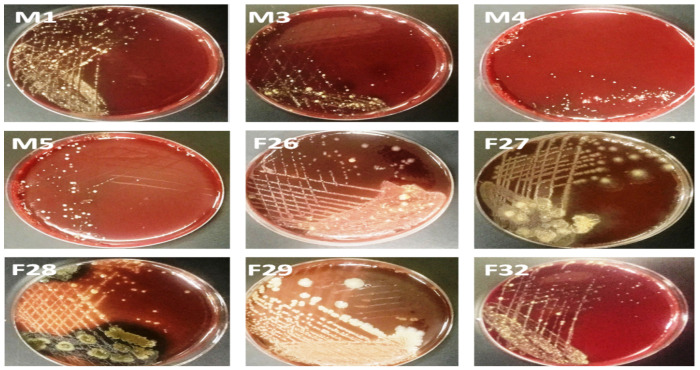
Bacterial growth on blood agar plates from sub-gingival plaque samples of males (M) and females (F) patients

Figure 1 show bacterial growth on blood agar plates from sub-gingival plaque samples of males (M) and females (F) patients. Plates were incubated at 37°C under anaerobic condition for 5 days.

**Figure 2:**
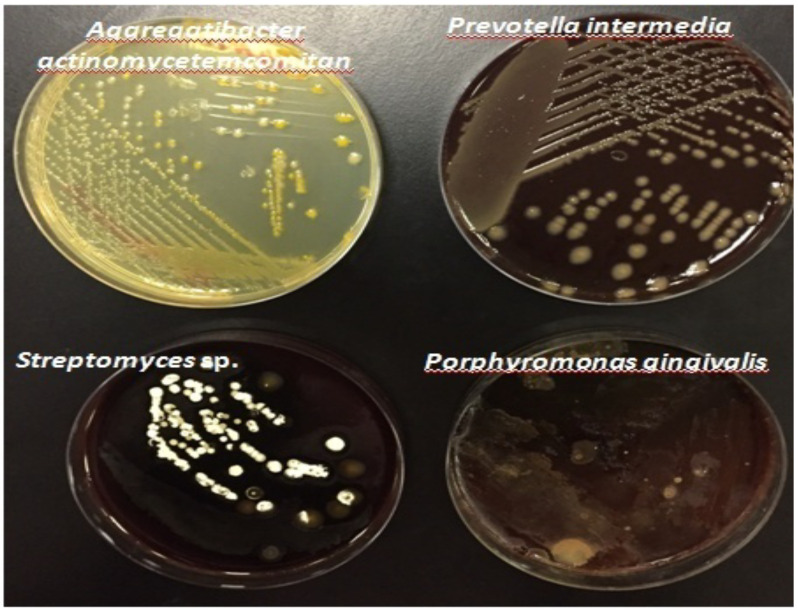
Bacterial growth (Pure colonies) of the main periodontal pathogens isolated from sub-gingival plaque samples of study group

Figure 2 shows bacterial growth (Pure colonies) of the main periodontal pathogens isolated from sub-gingival plaque samples of study group, incubated in blood agar plates at 37°C under anaerobic condition for 5 days.

- Aggregatibacteractinomycetemcomitan
- Prevotellaintermedia
- Porphyromonasgingivalis
- Streptomyces sp

**Figure 3:**
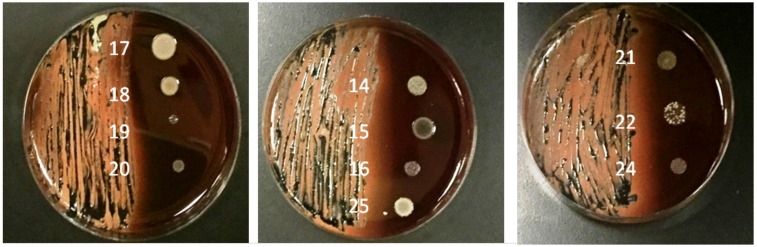
Effect of tar on some bacterial isolates from sub-gingival plaque samples of study Group

Figure 3 shows **e**ffect of tar on some bacterial isolates from sub-gingival plaque samples of study group. Each blood agar plate was divided into two halves: to the right, strains are cultured onto blood agar without tar (positive control) and to the left are the same strains cultured onto the section of the blood agar coated with tar. Note the inhibition of growth in all strains in the tar-coated sections.

**Figure 4:**
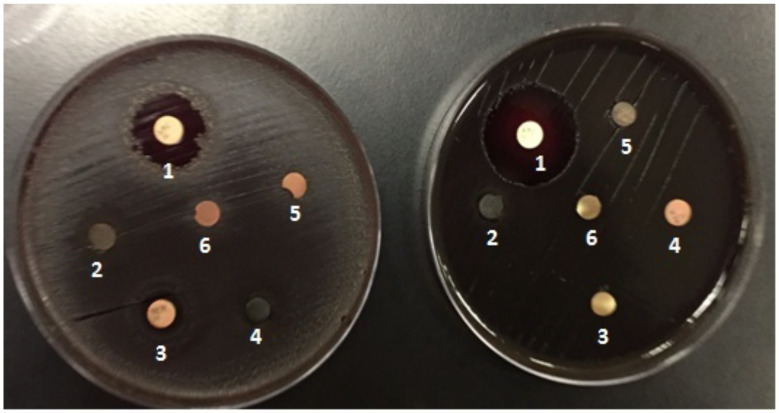
Determination of the MIC of tar on Aggregatibacteractinomycetemcomitan (A) and Porphyromonasgingivalis recovered from subgingival plaque sample in comparison to a positive control and a negative control

Figure 4 shows determination of the MIC of tar on *Aggregatibacteractinomycetemcomitan* (A) and *Porphyromonasgingivalis* recovered from subgingival plaque sample in comparison to a positive control and a negative control. Test was done on Mueller Hinton agar plates which were incubated at 37°C for 5 days. Abbreviations: 1 -amoxicillin (positive control); 2 - Tar 1/10 concentration; 3 - metronidazole; 4 - Tar original concentration; 5 - Tar 1/100 concentration; 6 - distilled water disk (negative control).

**Figure 5:**
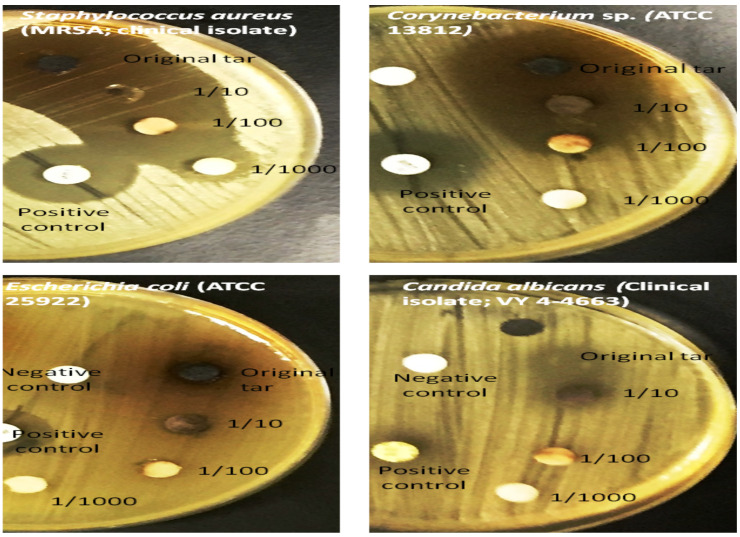
Determination of the MIC of tar on some bacterial and fungal sub-gingival plaque Samples

Figure 5 shows determination of the MIC of tar on some bacterial and fungal sub-gingival plaque samples. Test was done on Mueller Hinton agar plates which were incubated at 37°C for 5 days. The test was done with the original tar, then dilution 1/10, 1/100 and 1/1000; note inhibition zones under and around colonies). Negative control is a disk impregnated in sterile distilled water and positive control disks were imipenem (bacteria) and amphotoricin B (fungi).

Table 1 shows antimicrobial sensitivity testing (MIC) as measured by (Zone of Inhibition in mm) of *Aggregatibacteractinomycetemcomitan* and *Porphyromonasgingivalis* against Tar in comparison to positive and negative controls.

**Table 1:** Antimicrobial sensitivity testing (MIC)

Strain	Tar1 /10	Tar 1/100	Tar 1/1000	Amoxicillin Clavulanate (30µg)	Metroni dazole (30µg)	Negative control (disk in DW)
**Porphyromonasgingivalis**	15 mm	11 mm	8 mm	20 mm	12 mm	0 mm
**Aggregatibacteractinomycetemcomitans**	18 mm	13 mm	9 mm	30 mm	0 mm	0 mm

## Discussion

This study demonstrated the efficiency of Pine tars as a potential antimicrobial agent. In vitro assay was conducted to determine MIC against specific pathogens by serial dilution analysis and disc diffusion method. Microbial strains which are predominantly involved in root cause of periodontitis were tested. *Porphyromonasgingivalis,* a gram negative bacterium secretes gingipain an endopeptidase enzyme which is the key factor for manifestation of periodontal disease (11–14). The pathogen degrades matrix *metalloprotinease* (MMP), *fibronectin*, collagen resulting in severe periodontitis. *Prevotellaintermedia*, a gram negative bacteria thrives in anaerobic condition is predominantly found pathogen population in acute necrotizing ulcerative gingivitis and dental abscess.

*Aggregatibacteractinomycetemcomitans*, a gram negative bacterium responsible for localized aggressive periodontitis. These are the pathogens that are highly populated in tissue microbial samples of chronic periodontitis. *Streptomyces*, a gram positive actinobacteria is commonly prevalent in dental tooth infection because of its spore forming ability (15–19).

*P.gingivalis, P.intermedia, Aggregatibacteractinomycetemcomitans* was cultured as per the standard protocol. The microbial were incubated with pine tar gel. After 24 hours incubation, an inhibition zone of about 12–13 mm around the 75 µl well was observed in all the four species in the agar plates. As per the microbial nomenclature, an antibiotic or an anti-microbial agent is bent to have significant inhibitory effect on microbial reproduction and considered positive if the inhibition zone of 1 mm or more around the well was observed. In this study the pine tar exhibited an inhibition zone of 12 mm (20). The result of disc diffusion analysis was sufficient enough to illustrate that 75 µl tar oil solution produced growth inhibition of microbial strains. Serial dilution was conducted with tar oil against *P. gingivalis* and *P. intermedia, A. actinomycetemcomitans* species (7, 21). The study observed a significant sensitivity for up to six serial dilutions of the tar oil solution against *P.gingivalis* and *P.intermedia, A. actinomycetemcomitans* species whereas *Streptomyces* species recorded significant sensitivity for five serial dilutions of the gel. A scientific study is always important to establish the efficacy of a lethal dose to determine its anti-microbial property. In this study we postulate the efficiency of pine tar oil as a potential gum paint medicine for tropical application on infected dental gum tissues. Its usage in alternative medicinal practise for centuries, have proved its reliability and edible nature, any alternative medicine to bring in drug market needs a confirmatory and extensive repeated scientific clinical study. Based on these collected data, the drug is assembled for treatment plan (22, 23). This study is novel in terms of introducing pine tar as an antibiotic replacement for the management of dental periodontitis condition. The in vitro study of these four major dental plaques causing microbial species sustains the rationale for launching pine tar as gum paint product for dental caries. Furiga *et al.* studied the effect of a pine bark extract (PBE) against artificial oral biofilms (24). In a study by Sugimoto *et al* (25), Pycnogenol® (PYC) a standardized bark extract from French maritime pine (Pinus pinaster Aiton) was used to evaluate antibacterial inhibitory effects on alveolar bone resorption, a characteristic feature of periodontitis, induced by *Porphyromonas gingivalis* and osteoclast differentiation, in rat models. The results revealed that PYC may prevent alveolar bone resorption through its antibacterial activity against *P. gingivalis* and by suppressing osteoclastogenesis (24). In another study, human gingival fibroblasts (HGF cells) were employed to assess the cytotoxic potential of the *P. tropicalis* and *P. elliottii* resins (26).

As evident from the above mentioned studies, Pine tree and is natural derivatives have proven to be therapeutic and preventative agents for periodontal infections in various *in vitro* studies and can prove to be a promising product for dental infections in humans too. Pine tar oil a naturally derived product has gained interest for its edible, anti-septic, abundant property. It's easy in preparation and traditional medicinal practice has increased the research focus as a potential candidate for pharmaceutical purpose and periodontal treatment agents. Any small molecule or a plant based product to be used as a drug has to undergo a rigorous clinical testing procedure, to be proclaimed as a medication. Pine tar with attenuated phenol concentration can be used as a potential anti-microbial agent against periodontal pathogens considering its anti-bacterial and cytotoxic properties. Pine tar even though in usage for many years, contains no sufficient scientific study. A sustainable scientific proof in the form of *in-vitro* and *in-vivo* data is imminent for various form of pine tar to establish its anti-bacterial property.
